# The Impact of Team‐Based Ordering Workflows on Ambulatory Physician EHR Time, Order Volume, and Visit Volume

**DOI:** 10.1111/1475-6773.70038

**Published:** 2025-09-06

**Authors:** Nate C. Apathy, Alice S. Yan, A. Jay Holmgren

**Affiliations:** ^1^ Health Policy & Management School of Public Health, University of Maryland College Park Maryland USA; ^2^ Regenstrief Institute Indianapolis Indiana USA; ^3^ Division of Clinical Informatics and Digital Transformation, Director, Center for Clinical Informatics and Improvement Research, University of California – San Francisco, San Francisco, CA San Francisco California USA

## Abstract

**Objective:**

To analyze national rates of team‐based ordering and evaluate changes in key outcomes following adoption.

**Study Setting and Design:**

We conducted an observational pre‐post intervention‐comparison study of 249,463 ambulatory physicians across 401 organizations using the Epic EHR. Our intervention was the adoption of team‐based ordering, measured as the proportion of orders involving team support. Outcomes include active ordering time, overall EHR time, order volume, and visit volume among adopter physicians.

**Data Sources and Analytic Sample:**

We analyzed the distribution and trends in team‐based ordering rates from Epic Signal (September 2019–March 2022). We used multi‐variable regression in a difference‐in‐differences framework to evaluate changes in our outcomes among 115 adopters of team‐based ordering and 3115 non‐adopters. We defined adopters as physicians who demonstrated a one‐time shift from 0% of orders to a consistent non‐zero share of orders, and non‐adopters as those who demonstrated constant 0% teamwork for at least 18 months.

**Principal Findings:**

Across our study period, 26.2% of orders involved team support, with surgical specialists averaging greater team‐based ordering (43.1%) than primary care (22.2%) and medical specialists (23.0%). There was no association between team‐based ordering adoption and time spent ordering (−0.13 min/visit, 95% CI: [−0.48 to 0.22]) or total EHR time (−1.42 min/visit, [−3.79 to 0.95]). Adoption was associated with a 26.8% relative increase in order volume (0.47 orders/visit, [0.14–0.80]) and a 22.3% relative increase in visit volume (6.50 visits/week [2.81–10.19]).

**Conclusions:**

Team‐based ordering rates are relatively low, and new adoption of team‐based ordering was not associated with physicians' time spent ordering or in the EHR overall. Teamwork may facilitate substantial increases in both order and visit volume, but a greater level of team‐based ordering may be required to realize EHR time savings.

**Summary**

- What is known on this topic
  ○ Team‐based ordering workflows (i.e., non‐physician staff pending orders for physician signature) have been associated with reduced total EHR time and lower turnover risk.
  ○ The existing evidence base consists of single‐site studies lacking both causal design and focus on key ordering‐related outcomes.
  ○ National rates of team‐based ordering and the impacts of adopting such workflows are not well understood
- What this study adds
  ○ The national average rate of team‐based ordering in our sample was 26.2%, with surgical specialists averaging greater team‐based ordering (43.1%) than primary care (22.2%) and medical specialists (23.0%)
  ○ Adopter physicians increased order volume by 26.8%, primarily for non‐medication orders, and visit volume by 22.3%. Adopters' team‐based ordering rates were low (17.3%), potentially preventing EHR time savings.
  ○ Physicians, health care organizations, and policymakers should consider team‐based ordering rate targets of at least 25% to increase the likelihood of administrative time savings for physicians.

## 1 Introduction

In recent years, the demands placed on ambulatory physicians' time have increased to unsustainable levels, driving high rates of burnout, scaled‐back work effort, turnover, and departure [1, 2, 3, 4, 5]. The demands on physician time are myriad and include increasing expectations for patient volume [6], administrative and documentation requirements for prior authorization and reimbursement [7], an explosion in patient messages [8, 9, 10], and low‐usability, inefficient electronic health record (EHR) systems that frequently push physicians' work hours into the evenings [11, 12, 13]. As a result, numerous efforts have aimed to alleviate these challenges with team‐based approaches involving delegation and shared management of EHR‐based work including documentation, inbox management, and placing orders [14, 15, 16, 17, 18, 19].

While ordering occupies a smaller share of physicians' time relative to tasks like documentation [20, 21, 22, 23, 24, 25], it remains an important area of research focus for a number of reasons. Ordering is one of the few EHR work domains in which physicians can use standard EHR functionality to nearly fully delegate the administrative portion of the process and limit effort to “signing off” on orders placed or queued (a process called “pending”) by other members of the care team. Team‐based ordering workflows also support the autonomy of non‐physician clinicians, who can spend orders according to their own clinical judgment [26]. These orders can then be evaluated by the physician for their clinical appropriateness, thus encouraging all team members practice at the “top of their license.”

Early evidence on team‐based ordering workflows has shown that higher rates of team‐based ordering, assessed via the “teamwork for orders” (TW_ORD_) measure proposed by Sinsky et al. [27], are associated with reduced EHR time per visit [28], greater productivity, and reduced turnover [29, 30, 31]. Despite these promising early studies, important knowledge gaps exist that may impede broader use of team‐based ordering. Critically, studies of team‐based ordering to date have occurred in single sites affiliated with academic health systems, which may not generalize to ambulatory physicians nationally. These single‐site studies also leave evidence gaps with respect to average levels of team‐based ordering workflows in practice across the US, how the adoption of these workflows may vary across specialties, and how rates have evolved over time. These gaps limit the ability of policymakers, medical professional societies, and specialty societies to assess the potential for increased adoption to reduce EHR burden. For example, if team‐based ordering rates are already high or growing rapidly, further incentivizing team‐based ordering is unlikely to be necessary, but if adoption is low and concentrated in certain specialties, policy or regulatory action to encourage team‐based ordering workflows may be needed. Further, existing evidence is largely associational, and no studies have explicitly analyzed the impact of *adoption* (i.e., the shift from no ordering support to some ordering support), and no studies have precisely evaluated the impact of team‐based ordering workflows on the key first‐order outcome of interest: the amount of time physicians spend in the EHR on ordering tasks. As a result, policymakers, regulators, and professional organizations seeking to address EHR burden lack the empirical evidence to guide efforts to reduce physician administrative burden. Most importantly, there is little evidence to guide practicing physicians and health system leaders on the implementation of team‐based ordering as they weigh different options to reduce physician EHR burden in the context of finite resources.

To address these knowledge gaps, we sought to answer three research questions: (1) What is the rate of team‐based ordering overall among ambulatory physicians in the United States?; (2) How do team‐based ordering rates vary across specialties and over time; and (3) What is the impact of adopting team‐based ordering workflows on physician ordering time, EHR time, order volume, and visit volume? We hypothesize that the adoption of team‐based ordering will decrease physician time spent in the EHR overall, concentrated in ordering time specifically. We hypothesize that overall order volume will remain stable as the ordering task is delegated, and visit volume will increase as physicians have more time available, analogous to our team's prior research finding increased visit volume after the adoption of team‐based documentation workflows [19].

Our study is the first to establish national rates of team‐based ordering adoption and use by ambulatory physicians over time and across specialties, and the first to analyze the impact of newly adopting team‐based ordering workflows on policy‐relevant measures of physician EHR time, utilization, and patient access. Our findings can inform organizations pursuing EHR optimization and team‐based clinic workflow redesign by providing critical evidence on the likely impact of new or increased teamwork for ordering. Our study is also the first to rigorously evaluate a concrete burden reduction intervention that is directly relevant to ongoing efforts to reduce physician administrative burden from the National Academy of Medicine (NAM), the American Medical Informatics Association (AMIA), and the American Medical Association (AMA). Finally, this study can inform future policymaking from the Assistant Secretary for Technology Policy (ASTP) and the Centers for Medicare and Medicaid Services (CMS), both of which were instrumental in designing and implementing the computerized physician order entry (CPOE) requirements in the Meaningful Use (MU) incentive program and, later, Promoting Interoperability (PI).

## 2 Methods

### 2.1 Data Collection

Our data consists of physician‐month observations of EHR use activity metadata from US‐based ambulatory physicians using the Epic EHR system over a 36‐month study period (September 2019 to March 2022). This data was drawn from Epic Systems' Signal analytics platform. Epic is a leading EHR vendor, with 44% of the US ambulatory market share [32].

Epic's Signal analytics platform measures ambulatory physician usage of the EHR primarily for efficiency and training purposes. The metadata provides detailed measures of physician EHR use (e.g., time spent in various domains within the EHR, order volume) and of productivity (e.g., visit volume) [33]. These data also capture the proportion of orders involving support from non‐physician team members [27], providing the unique opportunity to identify and evaluate changes in outcomes for individual physicians following the adoption and onset of team‐based ordering workflows.

The data also includes physician specialty and organizational characteristics such as US Census Region, organizational type (i.e., ambulatory only, hospital and clinic, or other, such as retail clinic), and other organizational characteristics like teaching status and safety‐net status. The data were de‐identified and deemed exempt from IRB review with a waiver of informed consent.

### 2.2 Study Design

To achieve our first two research questions of understanding variations in team‐based ordering rates within our study population, we examined the distribution of team‐based ordering rates at the physician‐month level across six categories, based on natural cut points: 0% of orders with teamwork; (0%–25%); [25%–50%]; [50%–75%]; [75%–100%]; and 100%. We also plotted average monthly rates of TW_ORD_ by specialty group over time, using specialty mappings from the National Electronic Health Records Survey (NEHRS): (a) primary care; (b) medical; (c) surgical; and (d) other. Our study population for this objective consisted of all U.S. ambulatory physicians who were observed for at least 6 months during the 36‐month study period. Within this population, physician‐months were included if they involved at least 10 orders and at least five patient visits, and observations with outcome values above the 99th percentile were excluded to remove implausible outlier values. The final study population amounted to 249,463 physicians across 401 organizations, for a total of 5,346,315 physician‐month observations.

To achieve our third research question of understanding the relationship between team‐based ordering and our outcomes, we conducted both descriptive analyses in the national cohort described above and used a staggered adoption difference‐in‐differences study design to estimate the impact of team‐based ordering adoption, including event study regressions, to analyze changes in our outcomes following the adoption of team‐based ordering among a longitudinal cohort of U.S. ambulatory physicians during the 36‐month study period. Our study population for the difference‐in‐differences analysis consisted of an unbalanced panel of 3230 physicians, including 115 adopters and 3115 comparison physicians across 299 organizations, for a total of 88,475 physician‐month observations.

### 2.3 Team‐Based Ordering Workflows Adoption

We used physician‐month measures of EHR use that capture the proportion of orders that involved team support, often described as “pending” orders by non‐physicians for later physician sign‐off. We measured the proportion of orders in each month with any non‐physician contribution. We defined team‐based ordering *adopters* as physicians who demonstrated a one‐time shift in behavior during the 36‐month study period from 0% of orders to a consistent non‐zero share of orders and were observed for at least 18 months, including at least 6 months before and after adoption. This data‐driven approach ensures the strictest possible definition of team‐based ordering adoption and guarantees both no treatment reversal (i.e., “post‐adoption” months with zero team‐based ordering) and no exposure to team‐based ordering in the pre‐adoption period. Given within‐physician variability in team‐based ordering rates, this strict definition constrains our sample of adopters but strengthens the internal validity of our estimates. While we do not directly observe the specific practice changes made by individual adopter physicians in our sample, our definition of “adoption” captures several common scenarios. For example, our set of adopters would include a physician who newly employs or is provided with a medical assistant (MA), or a physician‐MA dyad that newly expands the scope of the MA (or other clinical team member) role to include order pending. Our comparison group consisted of physicians who demonstrated constant 0% teamwork throughout the sample period who were observed for at least 18 months. We excluded physicians with any team‐based ordering from this comparison group, as they are essentially previously treated units that constitute a “forbidden comparison” in the context of difference‐in‐differences analyses [34]. Inclusion of these physicians also led to violation of the parallel pre‐trends assumption required for causal inference in our context.

### 2.4 Outcome Measures

We analyzed the impact of team‐based ordering adoption on 6 key outcomes, all observed at the physician‐month level: active ordering time per visit, total EHR time per visit, total order volume per visit, total non‐medication orders, total medication orders, and average weekly ambulatory visit volume.

### 2.5 Statistical Analysis

For our first two research questions, we described the distribution of team‐based ordering rates at the physician‐month level across six categories: 0% of orders with teamwork; (0%–25%); [25%–50%]; [50%–75%]; [75%–100%]; and 100%. We also plotted a time series of average monthly rates of team‐based ordering by specialty group. Finally, we calculated the average of all outcomes across the six categories of team‐based ordering rates within the national sample. To estimate differences in the outcomes across categories and test the significance of these differences, we used ordinary least squares regression models adjusting for physician and time fixed effects.

For our third research question, we first calculated descriptive statistics overall and stratified across team‐based ordering adopters and non‐adopters for organization and physician characteristics. Next, we calculated average values for all outcome measures across three groups: adopters pre‐adoption, adopters post‐adoption, and comparison group physicians. To estimate average changes following the onset of team‐based ordering for adopting physicians (average treatment effect on the treated [ATT]), we ran a series of ordinary least squares multi‐variable regression models, using a two‐way fixed effects (TWFE) difference‐in‐differences approach. Each model regresses a given outcome on a binary variable set to 1 for adopter physicians in postadoption months and 0 in all other cases. All models include physician and month fixed effects to adjust for time‐invariant physician characteristics and secular trends, respectively. Estimates from these models reflect the average change in outcomes following team‐based ordering adoption among adopters, and these estimates do not generalize to non‐adopter physicians. Because adoption in our sample is spread across the study period and not concentrated in a few time periods, these estimates are at risk of bias derived from over‐weighting observations from early adopters and underweighting late adopters. To account for this risk, we re‐ran all models using the Callaway & Sant'Anna (CS) staggered treatment adoption regression approach specifying our cohort of never‐adopters as the comparison group [35]. This approach has been applied and explained further in similar studies of physician adoption of team‐based documentation approaches [19, 36, 37]. We favor these bias‐corrected estimates over standard TWFE estimates, so present both in the results but rely on the CS estimates for interpretation in the discussion.

Finally, to assess variation in changes following team‐based ordering over time, we used an event study regression approach that flexibly estimates monthly changes via an interaction term capturing months relative to treatment (−30 to 30, with adoption beginning at month 0) and a binary indicator for the treatment group (adopter or non‐adopter). This approach estimates the difference in our outcomes between adopters and comparison physicians in each month leading up to and following adoption. Estimates in the months following adoption allow us to assess variation in the ATT over time, while null estimates in the months prior to adoption support the assumption of parallel trends on which difference‐in‐differences inference relies (i.e., no trend differences in the preadoption period between adopters and nonadopters). All 2‐sided regression models used a cutoff of *p* < 0.05 for statistical significance, and we used heteroskedasticity‐robust standard errors clustered at the individual physician level to account for autocorrelation. All analyses were conducted in R statistical software, version 4.3.2 (R Project for Statistical Computing). Due to the use of de‐identified data, the University of Maryland Institutional Review Board deemed this study to be non‐human subjects research.

## 3 Results

### 3.1 Study Population

Our national sample of physicians consisted of 41.1% primary care physicians (*n* = 102,538), 32.9% medical specialists (*n* = 82,102), and 18.0% surgical specialists (*n* = 44,925). Physicians in this sample were predominantly from larger organizations, with 70.8% of organizations (*n* = 284) including more than 200 physicians. The national sample also included a majority of organizations that were classified as hospital and clinic facilities (82.3%, *n* = 330), with fewer ambulatory‐only organizations (14.2%, *n* = 57).

Our sample of physicians that adopted team‐based ordering workflows during the study period (*n* = 115) and the cohort of comparison physicians (*n* = 3115) consisted of 9.2% primary care physicians (*n* = 289), 68.7% medical specialists (*n* = 2247), 7.4% surgical specialists (*n* = 220), and 14.7% other specialists (*n* = 474). Unlike the national sample, physicians in our teamwork adoption sample were overwhelmingly from small organizations, with 89.7% of the organizations (*n* = 268) in this sample including fewer than 25 physicians. Most of these organizations were hospital and clinic facilities (86.3%, *n* = 258), with 9.0% classified as ambulatory‐only (*n* = 27). Additional sample statistics are available in Table 1.

**TABLE 1:** Characteristics of national and difference‐in‐difference samples, physician and organization‐level.

Characteristic	National sample	Difference‐in‐difference sample			
	Overall	Overall	Adopters pre‐adoption	Adopters post‐adoption	Comparison group
No. of physicians	249,463	3230	115	115	3115
No. of organization	401	299	68	68	294
No. of physician‐months	5,346,315	88,475	1324	1791	85,360
Percentage of orders with teamwork (mean, SD)	26.19 (29.94)	0.35 (3.76)	0.00 (0.00)	17.28 (20.15)	0.00 (0.00)
Primary outcomes, mean (SD)					
Time in EHR per visit (min)	17.22 (10.65)	20.35 (11.58)	17.48 (11.77)	16.01 (10.29)	20.49 (11.58)
Time in orders per visit (min)	2.35 (1.63)	2.39 (1.38)	2.19 (1.38)	2.12 (1.47)	2.39 (1.38)
Total orders per visit (count)	2.56 (1.82)	1.76 (1.48)	1.67 (1.38)	2.25 (1.82)	1.75 (1.47)
Total medication orders per visit (count)	0.66 (0.63)	0.95 (0.85)	0.69 (0.57)	0.68 (0.58)	0.96 (0.86)
Total non‐medication orders per visit (count)	1.90 (1.54)	0.80 (1.37)	0.99 (1.28)	1.56 (1.57)	0.78 (1.37)
Total visits per week (count)	37.35 (25.05)	29.21 (22.07)	33.01 (23.96)	39.18 (22.24)	28.94 (21.98)
Physician specialty (%)					
Primary CARE	41.1	9.2	17.4	17.4	8.6
Medical specialty	32.9	68.7	43.3	43.3	70.6
Surgical specialty	18.0	7.4	23.6	23.6	6.2
Other specialty	8.0	14.7	15.7	15.7	14.6
Organization characteristics (%)					
Size					
< 25 Physicians	1.0	89.7	74.9	74.9	89.4
25–50 Physicians	1.5	7.0	17.6	17.6	7.2
51–200 Physicians	26.7	3.3	7.5	7.5	3.4
> 200 Physicians	70.8	0.0	0.0	0.0	0.0
Type					
Hospital and clinic facilities	82.3	86.3	88.1	88.1	86.0
Ambulatory only	14.2	9.0	7.5	7.5	9.2
Other	3.5	4.7	4.4	4.4	4.8
Status					
Academic	23.4	30.1	35.3	35.3	30.6
Teaching	32.7	38.8	52.9	52.9	43.3
Pediatric	6.0	6.7	4.4	4.4	6.8
Safety Net	22.2	25.1	19.1	19.1	25.2
Community Hospital	32.2	33.1	35.3	35.3	32.3
Religiously Affiliated	8.7	8.7	5.9	5.9	8.8
US Census Region					
Midwest	25.9	27.4	22.2	22.2	27.5
Northeast	18.7	22.1	33.4	33.4	22.1
South	30.2	26.1	22.2	22.2	25.9
West	25.2	24.4	22.2	22.2	24.5

*Note:* Outcome averages are calculated for all physician‐months.

Abbreviations: EHR, electronic health record; SD, standard deviation.

### 3.2 Rates of Team‐Based Ordering

**FIGURE 1:**
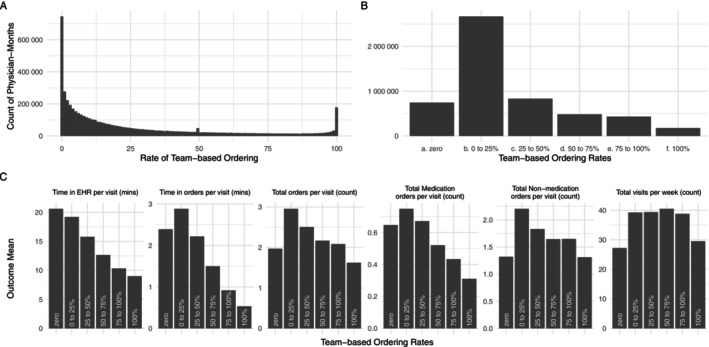
Distribution of team‐based ordering, September 2019–April 2022. (A) The distribution of rates of team‐based ordering (percentage of orders with team contribution) observed at the physician‐month level. Each bar contains a single percentage point, from 0% to 100% of orders. (B) The distribution of rates of team‐based ordering categorized into 25 percentage‐point categories. In both panels, the y‐axis is the count of physician‐months. (C) The outcome means for all six outcomes across categories of team‐based ordering rates. Medication and non‐medication orders sum to total orders.

Nationally, the average rate of team‐based ordering was 26.2% of orders (SD: 29.9, median: 13.0%); however, the rate of team‐based ordering was not normally distributed (Figure 1A). 14.0% of physician‐months in the national sample involved zero teamwork for ordering, and for 3.3% of physician‐months, all orders involved teamwork. The plurality (49.9%) of physician‐months included more than zero but less than a quarter of orders with any teamwork (Figure 1B). Finally, we found differences in all outcomes across rates of team‐based ordering (Figure 1C, Table S1). Overall EHR time decreased with higher rates of team‐based ordering, but clinically meaningful declines were only seen when at least 25% of orders involved teamwork. Per‐visit time spent on ordering tasks increased significantly when more than zero but 25% of orders involved teamwork, relative to physician‐months with zero team‐based orders. With this outcome, as well, decreases only occurred beyond 25% of orders with teamwork.

### 3.3 Variation in Team‐Based Ordering Across Specialties and Over Time

**FIGURE 2:**
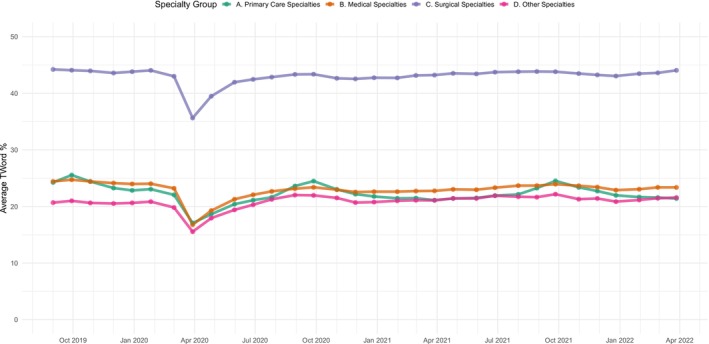
Rates of team‐based ordering across specialties, September 2019–April 2022. Lines show average rates of team‐based ordering (percentage of orders with team contribution) for each specialty group in each month of the study period. Specialty groups are defined using specialty categorizations from the National Electronic Health Records Survey (NEHRS).

Specialties varied in their rates of team‐based ordering, with surgical specialists exceeding other specialty groups. On average, surgical specialists had teamwork for 43.1% (SD: 36.3) of orders, compared to 22.2% (SD: 23.6) for primary care specialists and 23.0% (SD: 30.2) for medical specialists. In examining trends in team‐based ordering over time, we found that rates were generally stable over time, with a decrease during the acute COVID‐19 pandemic onset period of March 2020 to June 2020 (Figure 2). While surgical specialists had the highest rates of teamwork for orders, these physicians had substantially lower overall volume of orders compared to primary care physicians and medical specialists, across all levels of team‐based ordering (Figure S1). Furthermore, surgical specialists predominantly placed non‐medication orders, while medication orders comprised a larger share of primary care and medical specialist orders (Figure S2).

### 3.4 Average Changes Following the Adoption of Team‐Based Ordering Workflows

In our analysis of physicians who adopted team‐based ordering workflows during our study period, standard TWFE difference‐in‐differences regressions illustrated a significant association between team‐based ordering adoption and all outcomes. Adoption of team‐based ordering was associated with less time in the EHR overall (B: −2.36 min per visit, 95% CI: −3.32 to −1.40), less time spent on ordering tasks in the EHR (B: −0.15 min per visit, 95% CI: −0.28 to −0.01), greater order volume (B: 0.62 orders per visit, 95% CI: 0.41–0.83) for both medication orders (TWFE EST) and non‐medication orders (TWFE EST), and greater visit volume (B: 6.69 visits per week, 95% CI: 4.11–9.28) (Table 2).

**TABLE 2:** Association of team‐based ordering adoption with outcomes, difference‐in‐differences estimates.

Outcome	TWFE estimate and 95% CI	CS ATT estimate and 95% CI	Outcome mean	Relative change
Time in EHR per visit (min)	−2.36 [−3.32, −1.4]***	−1.42 [−3.79, 0.95]	20.4	—
Time in orders per visit (min)	−0.15 [−0.28, −0.01]*	−0.13 [−0.48, 0.22]	2.39	—
Total orders per visit (count)	0.62 [0.41, 0.83]***	**0.47 [0.14, 0.80]**	1.76	+26.8%
Total medication orders per visit (count)	0.05 [0.01, 0.09]*	**0.10 [0.01, 0.19]**	0.95	+10.5%
Total non‐medication Orders per visit (count)	0.57 [0.36; 0.78]***	**0.37 [0.05, 0.69]**	0.80	+46.3%
Total visits per week (count)	6.69 [4.11, 9.28]***	**6.50 [2.81, 10.19]**	29.2	+22.3%

*Note:* *p < 0.05; **p < 0.01; ***p < 0.001. Relative changes are calculated based on estimates from CS regression models. CS models do not yield precise *p* value estimates; thus, statistical significance is determined based on whether the 95% CI includes zero. Bold value represents a statistically significant *p*‐value, meaning *p* < 0.05.

Abbreviations: CI, confidence interval; CS ATT, Callaway & Sant'Anna average treatment effect on the treated; EHR, electronic health record; TWFE, two‐way fixed effects.

However, as noted above, TWFE estimates are likely to be biased, and our estimates derived from the CS approach rendered the EHR time‐based outcome estimates null (Table 2). We favor these bias‐corrected CS estimates that illustrated no association between adoption of team‐based ordering workflows and overall EHR time (B: −1.42 min per visit, 95% CI: −3.79 to 0.95) or time spent on ordering (B: −0.13 min per visit, 95% CI: −0.48 to 0.22). However, CS estimates confirmed an association between team‐based ordering adoption and a 26.8% relative increase in order volume (B: 0.47 orders per visit, 95% CI: 0.14–0.80)—concentrated in non‐medication orders (B: 0.37 non‐medication orders per visit, 95% CI: 0.05–0.69)—along with a 22.3% relative increase in visit volume (B: 6.50 visits per week, 95% CI: 2.81–10.19) (Table 2).

**FIGURE 3:**
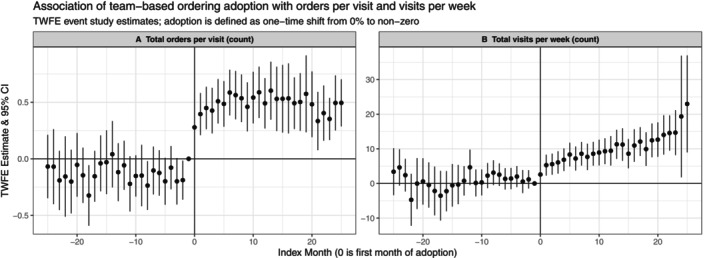
Association of team‐based ordering adoption with order and visit volume. Differences over time in outcomes between physicians who adopted team‐based ordering workflows and those who had no team‐based ordering throughout the study period. Estimates are from two‐way fixed effects event study regression models with indicators for each month preceding and following adoption, indicators for adopters, and the interaction between these two terms. Data points indicate coefficient estimates, and bars represent the 95% confidence intervals of the differences in outcomes between adopters and non‐adopters leading up to and after support adoption. The vertical dashed lines represent the first week of team‐based ordering among adopters, with Month 1 serving as the reference month for estimates in each panel. Month 0 on the x‐axis is the first observed month of team‐based ordering adoption. The unit of observation is the physician‐month. Plots of the association of team‐based ordering adoption with total active EHR time, ordering time, and order type composition are available in Figure S3.

Event study estimates illustrated largely null estimates in the pre‐adoption period for all primary outcomes, consistent with the parallel pre‐trends assumption required for causal inference using difference‐in‐differences. While we interpret our estimates as associations, the general lack of non‐zero coefficient estimates in the pre‐adoption period lends strength to the internal validity of our estimates and the attribution of those changes to the adoption of team‐based ordering workflows rather than any other concomitant change. Event studies showed a sharp increase in order volume at the time of adoption onset with only minor variation over time in the magnitude of the estimates (Figure 3A). Visit volume, however, did illustrate an increasing magnitude of estimates over time, suggesting that visit volume continued to increase over time for adopters (Figure 3B). For example, in the second month following the adoption of team‐based orders, adopters had on average increased visit volume by 5.3 visits per week (95% CI: 2.3–8.3). By month 24, that average change had increased to 14.7 additional visits per week (95% CI: 8.2–21.2). Event study plots for order composition, overall EHR time, and ordering time outcomes are available in Figure S3.

## 4 Discussion

Using a unique national dataset, we found national rates of team‐based ordering workflows among ambulatory physicians were generally low. An average of 26% and a median of 13% of orders had contributions from non‐physician members of the care team, but on average, well over half of orders are placed fully by physicians, even among surgical specialists. Average rates have remained stable over time, suggesting that current practices may reflect an equilibrium rate of team‐based ordering between 10% and 25% for most physicians; however, our data do not allow us to identify the “optimal” rate of team‐based ordering, which likely differs by specialty and across physicians [23, 38, 39]. Furthermore, team stability may play an important role in team‐based ordering practices in particular, as longer‐tenured staff can take on an increasing amount of ordering responsibilities for a clinic over time. High rates of clinician turnover may prevent this knowledge from accruing and, as a result, place a ceiling on rates of team‐based ordering. Further examination, both quantitative and qualitative, of the factors that enable consistently high rates of teamwork for ordering workflows is needed to fully understand optimal rates of team‐based ordering and the facilitators of those workflows. This is especially important given our descriptive findings that administrative EHR time savings appear to only occur for physicians when at least 25% of orders are placed with teamwork (Figure 1C).

Contrary to our expectations, our difference‐in‐differences analysis found no evidence of per‐visit time savings in the EHR overall or specifically on ordering tasks for physicians who newly adopted team‐based ordering workflows (Table 2). This finding also contrasts with prior studies that have found significant time savings for adopters of teamwork for documentation, conditional on team members taking on a sufficient proportion of documentation [19]. The most likely explanation for this finding is the relatively low post‐adoption rates of team‐based ordering in this sample (17.3%), which is below the national average of 26.2% and below the potential threshold rate of 25% that is associated with clinically meaningful EHR time savings in the national sample (Figure 1C and Table S1). This suggests there may be a “minimum viable” amount of teamwork that must occur for physicians to realize clinically meaningful time savings on ordering tasks. On the other hand, unlike with documentation, ordering occupies a quite small portion of physicians' time in the EHR [25], and the fixed time costs of ordering may impose a floor below which physician ordering time cannot reasonably be expected to go.

It also stands to reason that the increase in total order volume that we observe counteracts any potential ordering time savings at the visit level. Our null estimate for change in ordering time coupled with this increase in total order volume definitionally implies a decrease in the amount of time physicians spend in the EHR *per order*, but does not allow us to explore the underlying composition of how that time is spent. For example, it is possible that after implementing team‐based ordering practices, a physician shifts her ordering time to entering and managing relatively complex orders while team members execute routine or protocol orders. In this scenario, this physician's active ordering time would not change, despite the nature of her ordering work changing substantially. However, our data only differentiates between medication and non‐medication orders, and does not contain more specific information about these additional orders, limiting our ability to assess how clinical care delivery changes following the adoption of team‐based ordering workflows. Given that prior to adoption, adopters placed fewer orders per visit than the national average, it may be that team‐based ordering brought these adopters up to parity with the national average, where a non‐zero amount of teamwork for orders is very common (Figure 2). It may be that physicians with no team‐based ordering were under‐provisioning care due to time constraints relative to their peers with some support, and that team‐based ordering will therefore increase care completeness and quality, especially given our finding that the vast majority of additional orders are non‐medication orders (e.g., laboratory tests, procedures, and referral orders). This is consistent with prior research establishing that team‐based ambulatory care is associated with increased care quality, as measured via outcomes like appropriate lab monitoring that are directly downstream of ordering practices [40]. Further research leveraging EHR use metadata is required to understand the downstream impact on the comprehensiveness and cost of care, following from changes to order volume and the mix of orders placed in ambulatory encounters following the introduction of team‐based ordering.

Finally, we observe a large increase in visit volume following the adoption of team‐based ordering workflows. This increase grows in magnitude over the post‐adoption period, up to nearly 15 additional visits per week 2 years into physicians' collaborative ordering experience, a 50% relative increase. It is unlikely that this magnitude of impact is attributable solely to a collaborative ordering process, given how little time ordering occupies relative to other tasks. Our exposure measure, therefore, may be measuring a broader phenomenon of clinical teamwork, additional investments in care teams, and/or additional hiring of support with revenue from increased volume. Our evidence suggests that consistent teamwork over time yields large gains in productivity for a given physician or clinic. EHR use metadata is particularly well‐suited to capture clinical teamwork in practice [41, 42, 43, 44, 45], and future research should leverage this data to explore the evolution of clinical teams and teamwork over time to better understand the mechanisms underlying the changes we observe. While our study has strengths in that we leverage a large nationwide sample of physicians with granular tracking of all EHR‐based activities, analyze outcomes across multiple diverse organizations, and employ a study design that minimizes bias from confounding, it also has important limitations. Primarily, the characteristics of our sample of team‐based ordering adopters differ from the national sample substantially (Table 1). This limits our ability to generalize our findings to physicians broadly, while still preserving the reliability of our ATT estimates, which are derived from comparison to never‐adopters. However, it is possible that the sample of adopter physicians is largely composed of “late adopters,” meaning that our results would generalize to other physicians currently deliberating on whether to adopt team‐based ordering workflows. Second, our adopter sample is relatively small, and adoption is spread out over our study period relatively evenly, which constrains the power of our analyses and could be contributing to the null CS estimates we recover for changes in EHR time and ordering time. Finally, we interpret all estimates as associational rather than causal given these limitations.

## 5 Implications for Policy and Practice

Our findings offer concrete guidance for physicians, health system leaders, and policymakers aiming to reduce physician EHR burden and improve practice efficiency. First, we find descriptively that physicians with more than 25% of orders placed via team‐based ordering spend less time in the EHR on ordering tasks than physicians with no team‐based ordering (Figure 1C). This offers a potential avenue, specifically formalizing team‐based ordering workflows in ambulatory contexts, for practice changes to reduce EHR‐based administrative burden on physicians. While our difference‐in‐differences analysis finding no impact on EHR time or ordering time may appear to contradict this guidance, it is crucial to note that within our adopter sample, the post‐adoption average for the rate of team‐based ordering was only 17.3% of orders (Table 1). This is below the 25% threshold identified in descriptive analysis, emphasizing that there is likely a minimum rate of team‐based ordering to achieve clinically meaningful time savings. Taken together, these findings reinforce the conclusion that simply adopting team‐based ordering may not be enough—a minimum intensity of team‐based ordering is required to reduce physician time spent on orders. For policymakers, the thresholds we identify could be incorporated into evidence‐based measures required for existing federal reporting programs like the Centers for Medicare and Medicaid Services' (CMS) Merit‐based Incentive Payment System (MIPS) Promoting Interoperability component. For example, attestation to a theoretical “teamwork for orders” metric could require physicians to report yes or no to “using team‐based ordering for at least 25% of orders,” with EHR metadata measures readily available to support these attestations. Another option could be to simply report the physician's rate of team‐based ordering during the reporting period, analogous to historical past measures of interoperability (e.g., “share of referrals sent with electronic summaries of care” from Meaningful Use Stage 2) [46, 47].

In conclusion, our study of national rates of team‐based ordering workflows illustrated relatively low overall rates of team‐based ordering. This finding suggests opportunities for primary care and medical specialist physicians in particular to increase team‐based ordering to rates above the time‐saving threshold of at least 25%. While our difference‐in‐differences estimates revealed no association between new adoption of these workflows and time spent on ordering tasks or in the EHR, it is possible that greater rates of team‐based ordering would lead to decreases in these outcomes. Given that new adopters averaged substantially fewer orders with teamwork than the national sample, organizations implementing team‐based ordering workflows should consider targets for team‐based ordering post‐implementation that are at or above the national average rate of 26%. Furthermore, quality improvement and federal incentive programs that require attestation to measures of physician order entry (e.g., ePrescribing in Promoting Interoperability) should consider including rates of team‐based ordering in these measure.

## Conflicts of Interest

The authors declare no conflicts of interest.

## Supporting information


**Appendix S1:** Supporting Information.

## Data Availability

The data that support the findings of this study are available from Epic Systems Corp. Restrictions apply to the availability of these data, which were used under license for this study. Data are available from the authors with the permission of Epic Systems Corp.
