# Supplementary material for: The Impact of Team‐Based Ordering Workflows on Ambulatory Physician EHR Time, Order Volume, and Visit Volume
Source: Health Serv Res. 2025 Sep 6;61(1):e70038. doi: 10.1111/1475-6773.70038 (PMC12857449; doi:10.1111/1475-6773.70038)
Supplement: Supplementary file 1 — Appendix S1: Supporting Information. [file HESR-61-0-s001.docx]

Supplementary Appendix for

*The impact of team-based ordering workflows on ambulatory physician EHR time, order volume, and visit volume*

**Contents**

Appendix Table 1. Two-way fixed-effects estimates of the relationship between team-based ordering rates and key outcomes, national sample

Appendix Figure 1. Median monthly order volume by rate of team-based ordering

Appendix Figure 2. Order type composition by physician specialty group

Appendix Figure 3. Association of team-based ordering adoption with active EHR time, ordering time, and order type composition

**Appendix Table 1. Two-way fixed-effects estimates of the relationship between team-based ordering rates and key outcomes, national sample**

|  | *Time in EHR per visit (mins)* | *Time in Orders per visit (mins)* | *Total Orders per visit (count)* | *Total Medication Orders per visit (count)* | *Total Non-Medication Orders per visit (count)* | *Total Visits per week (count)* |
| --- | --- | --- | --- | --- | --- | --- |
|  | B & 95% CI | B & 95% CI | B & 95% CI | B & 95% CI | B & 95% CI | B & 95% CI |
| Team-based ordering rates | |  |  |  |  |  |
| 0% of orders | ref | ref | ref | ref | ref | ref |
| 0 to 25% | -0.37***  [-0.39; -0.34] | 0.01***  [0.01; 0.02] | 0.17***  [0.16; 0.17] | 0.01***  [0.01; 0.01] | 0.16***  [0.15; 0.16] | 1.15***  [1.10; 1.20] |
| 25 to 50% | -1.17***  [-1.20; -1.13] | -0.16***  [-0.17; -0.16] | 0.27***  [0.27; 0.28] | 0.02***  [0.01; 0.02] | 0.26***  [0.25; 0.26] | 1.78***  [1.70; 1.86] |
| 50 to 75% | -2.10***  [-2.14; -2.05] | -0.38***  [-0.39; -0.37] | 0.35***  [0.34; 0.36] | 0.01***  [0.00; 0.01] | 0.34***  [0.33; 0.35] | 2.29***  [2.17; 2.42] |
| 75 to 100% | -3.09***  [-3.15; -3.03] | -0.62***  [-0.64; -0.61] | 0.46***  [0.44; 0.47] | -0.01*  [-0.01; 0.00] | 0.46***  [0.45; 0.48] | 2.67***  [2.50; 2.85] |
| 100% | -3.47***  [-3.55; -3.40] | -0.73***  [-0.74; -0.72] | 0.38***  [0.36; 0.40] | -0.04***  [-0.04; -0.03] | 0.41***  [0.39; 0.43] | 1.50***  [1.28; 1.71] |
| outcome mean (sd) | 17.22 (10.65) | 2.35 (1.63) | 2.56 (1.82) | 0.66 (0.63) | 1.90 (1.54) | 37.35 (25.05) |
| observations | 5,346,315 | 5,346,315 | 5,346,315 | 5,346,315 | 5,346,315 | 5,346,315 |

*Notes: Estimates are from two-way fixed-effects regressions adjusting for physician and month fixed-effects. Errors are clustered at the physician level. *p<0.05; **p<0.01; ***p<0.001.*

**Appendix Figure 1. Median monthly order volume by rate of team-based ordering**

**Appendix Figure 2. Order type composition by physician specialty group**

**Appendix Figure 3. Association of team-based ordering adoption with active EHR time, ordering time, and order type composition**
